# Antifungal and Antivirulence Activity of Vaginal *Lactobacillus* Spp. Products against *Candida* Vaginal Isolates

**DOI:** 10.3390/pathogens8030150

**Published:** 2019-09-12

**Authors:** Camilla Itapary dos Santos, Yasmine Ramos França, Carmem Duarte Lima Campos, Maria Rosa Quaresma Bomfim, Bruna Oliveira Melo, Rodrigo Assunção Holanda, Vera Lucia Santos, Sílvio Gomes Monteiro, Eduardo Buozzi Moffa, Andrea Souza Monteiro, Cristina Andrade Monteiro, Valério Monteiro-Neto

**Affiliations:** 1 Laboratório de Microbiologia Aplicada, Programa de Mestrado em Biologia Parasitária, Universidade Ceuma, São Luís, MA 65075120, Brazil; camilla_itapary@hotmail.com (C.I.d.S.); minyfranca@gmail.com (Y.R.F.); 2 Programa de Mestrado em Biologia Microbiana, Universidade Ceuma, São Luís, MA 65075120, Brazileduardo.moffa@ceuma.br (E.B.M.); andreasmont@gmail.com (A.S.M.); valerio.monteiro@ceuma.br (V.M.-N.); 3 Laboratório de Biologia Molecular de Microrganismos, Programa de Mestrado em Biologia Microbiana, Universidade Ceuma, São Luís, MA 65075120, Brazilbrunaoliv96@gmail.com (B.O.M.); raholanda@yahoo.com.br (R.A.H.); 4 Laboratório de Microbiologia Aplicada, Departamento de Microbiologia, Instituto de Ciências Biológicas, Universidade Federal de Minas Gerais, Belo Horizonte, MG 31270901, Brazil; verabio@gmail.com; 5 Centro de Ciências Biológicas e da Saúde, Universidade Federal do Maranhão, São Luís, MA 65080-805, Brazil; silvio_gm@yahoo.com.br; 6 Programa de Mestrado em Meio Ambiente, Universidade Ceuma, São Luís, MA 65075120, Brazil; 7 Departamento de Biologia, Instituto Federal do Maranhão, São Luís, MA 65030005, Brazil

**Keywords:** *Lactobacillus* biosurfactant production, biofilm inhibition, *Candida albicans* suppression, *Lactobacillus* antivirulence, vulvovaginal candidiasis

## Abstract

*Candida* yeasts are generally found in the vaginal microbiota; however, disruption of the balance maintained by host factors and microorganisms results in vulvovaginal candidiasis (VVC). This study evaluated the antagonistic activity of vaginal *Lactobacillus* spp. on *Candida albicans* to verify whether active compounds of *Lactobacillus* spp. had antifungal and antivirulence activity. The antagonism assay showed that 15 out of 20 *Lactobacillus* strains had an inhibitory effect on *C. albicans.* Biosurfactants displayed surface-tension-reducing activity, with the best value obtained for *Lactobacillus gasseri* 1. *Lactobacillus rhamnosus* ATCC 9595, *Lactobacillus acidophilus* ATCC 4356, and *Lactobacillus paracasei* 11 produced biosurfactants that decreased *C. albicans* adhesion and disrupted biofilm formation. The best results were obtained in the pre-incubation assay for *L. gasseri* 1 and *L. paracasei* 11. Overall, *Lactobacillus* strains showed significant anti-*Candida* activity, and their biosurfactants exhibited considerable anti-adhesion and antibiofilm activity against *C. albicans*. To be considered safe for use in vivo, the safety of biosurfactant (BS) should be investigated using cytotoxicity assays.

## 1. Introduction

*Candida* yeasts are generally found in the vaginal microbiota, but their presence does not always lead to the manifestation of symptoms. The complex interactions and synergies among host defense mechanisms and different microorganisms from the vaginal mucosa are responsible for maintaining the balance of the vaginal environment [1]. 

When homeostasis of the vaginal ecosystem is interrupted, overgrowth of *Candida* yeasts is facilitated and can lead to the development of vulvovaginal candidiasis (VVC) [2]. Primary symptoms of VVC are itching and soreness of the vulva, dysuria, white vaginal discharge, and dyspareunia. VVC can greatly affect the quality of life, in addition to increasing human immunodeficiency virus (HIV) susceptibility [3]. Although VVC is associated with a very low mortality rate, symptoms contribute significantly to morbidity, especially in HIV-infected women. [4]. Furthermore, women with vaginal colonization of *Candida* spp. during the second trimester of pregnancy have lower neonatal birth weight and higher rates of preterm birth than those colonized during other months of pregnancy [5].

Factors that increase the risk for VVC development include individual susceptibility, frequent sexual intercourse, antibiotic therapy, contraceptive and spermicide use, pregnancy, diabetes, and immunosuppression [3]. VVC is most commonly caused by *Candida albicans*, but the incidence of VVC caused by other *Candida* spp. has increased considerably [6,7,8]. Species, such as *Candida glabrata*, *Candida parapsilosis, Candida krusei*, and *Candida tropicals* are isolated with increasing frequency [7,8,9,10]. Increased infections by other *Candida* spp. have contributed to high rates of recurrence and resistance [10,11,12,13].

*Lactobacillus* spp. are considered normal colonizers of the human body, forming a part of the resident microbiota, and do not damage the host. In healthy vaginal microbiota, *Lactobacillus* spp. are one of the most abundant microorganisms [14]. *Lactobacillus* spp. control the excessive multiplication of potential pathogens by producing organic acids and antimicrobial compounds (hydrogen peroxide, bacteriocins, and surface-active compounds, including biosurfactants (BSs)), by auto-aggregation, or by competing for nutrients and adherence sites in the vaginal epithelium [15,16,17]. However, the pathogenesis of VVC remains a controversial issue. Individual susceptibility (genetics), pregnancy, antibiotic therapy, use of contraceptives and spermicide, frequent sexual intercourse, diabetes, and immunosuppression are factors that increase the risk for development of VVC [2,18].

Microorganisms can synthesize several types of surface-active compounds, including BSs, which have low molecular weights. BSs exhibit surfactant and emulsifying activity, and, therefore, have the ability to decrease the interface between two phases of a heterogeneous system; besides, they are useful as antibacterial, antifungal, anti-adhesive, and antibiofilm agents, and even have potential for use as major immunomodulatory molecules or in vaccines and gene therapy [19].

Probiotics are defined as “live microorganisms that, when administered in adequate amounts, confer a health benefit to the host” [20]. Several studies have reported the potential use of BSs produced by lactic acid bacteria (LAB) in the food and health industries [21,22,23,24,25]. In the food industry, it can be used as a treatment of food-contact surfaces, thus preventing biofilm formation; food additive/ingredient, and in residues treatment [26]. Their potential use in health industries may be related with anti-adhesive properties, which inhibit the adhesion of pathogenic organisms to solid surfaces, such as silicone rubber, surgical implants, and vinyl urethral catheters, or biological surfaces (urogenital and intestinal tract epithelial cells) [25,27,28]. Besides, BS may be used in pharmaceutical fields as agents for respiratory failure, immunological adjuvants, recovery of intracellular products, antimicrobial activity, antiviral activity, anticancer activity, and agents for the stimulation of skin fibroblast metabolism [29]. LAB interference in pathogen colonization occurs through multiple mechanisms, including BS production [30]. 

Surface-active compounds could be an alternative method to interfere with or avoid colonization by pathogenic microorganisms, preventing the progression of infections. Recently, *Lactobacillus* spp. have attracted the attention of the medical community due to their antagonistic effects against innumerable human pathogens, indicating potential therapeutic or prophylactic use for certain infectious diseases [31,32]. We have recently shown that *Lactobacillus fermentum* ATCC 23271 displayed antagonistic activity on *Candida* species in vitro and also inhibited yeast adherence to HeLa cells and mucin [33]. However, the effects and anti-*Candida* mechanisms of *Lactobacillus* BSs are still not fully understood, especially those related to resident *Lactobacillus* spp.

This work aimed to evaluate the antagonistic activity of *Lactobacillus* spp. from vaginal specimens on *C. albicans* from healthy women and those with clinical suspicion of VVC, verifying whether the active compounds of *Lactobacillus* spp., including BSs, have antifungal activities, and whether they interfere with the adhesion and biofilm processes of *Candida albicans*. A major contribution of this work was the identification of natural *Lactobacillus* species from the microbiota that have probiotic potential against *Candida* species.

## 2. Results

### 2.1. Microbial Isolation and Identification

Vaginal specimens were collected from 50 patients between 18 and 79 years of age during a colpocytology examination at a public hospital in São Luís, Maranhão, Brazil. From these samples, 16 *Candida* and 15 *Lactobacillus* isolates were detected (Table 1). In specimens of certain patients, concomitant detection of *Candida* and *Lactobacillus* isolates occurred in the same sample. Six *Candida* spp. and seven *Lactobacillus* spp. were isolated from 11 asymptomatic women. *C. albicans* (50%) and *Lactobacillus gasseri* (50%) were the most prevalent species in this patient group. Ten *Candida* spp. and eight *Lactobacillus* spp. were isolated from 14 women with VVC. The most frequent yeasts of the VVC group were *C. albicans* (50%) and *C. glabrata* (50%), while *L. gasseri* was the most frequent *Lactobacillus* species (57%). There was no difference in the frequency of *Candida* spp. between asymptomatic patients and those with VVC. The same was observed for *Lactobacillus* spp. We have adopted molecular identification methods as the standard for yeasts and bacteria.

### 2.2. Antagonism Assay

The antagonism assay showed that 15 of 20 *Lactobacillus* strains, including the five reference strains, had an inhibitory effect on *C. albicans*. Growth inhibition zones ranged from 9.5 to 28.5 mm (Table 2). Among the antagonistic strains, *L. acidophilus* ATCC 4356, *L. rhamnosus* ATCC 9595, and the clinical isolate *L. paracasei* 11 (Lp11) were able to inhibit all *Candida* studied. Evaluation by Tukey’s statistical test showed that the inhibitory ability was dependent upon both the *Candida* and *Lactobacillus* strains tested.

The anti-*Candida* activity of *Lactobacillus* from asymptomatic women was higher than that of *Lactobacillus* from patients with suspected VVC (Table 3; *p* < 0.05).

### 2.3. BS-Producing Lactobacilli and BS Properties

**Figure 1:**
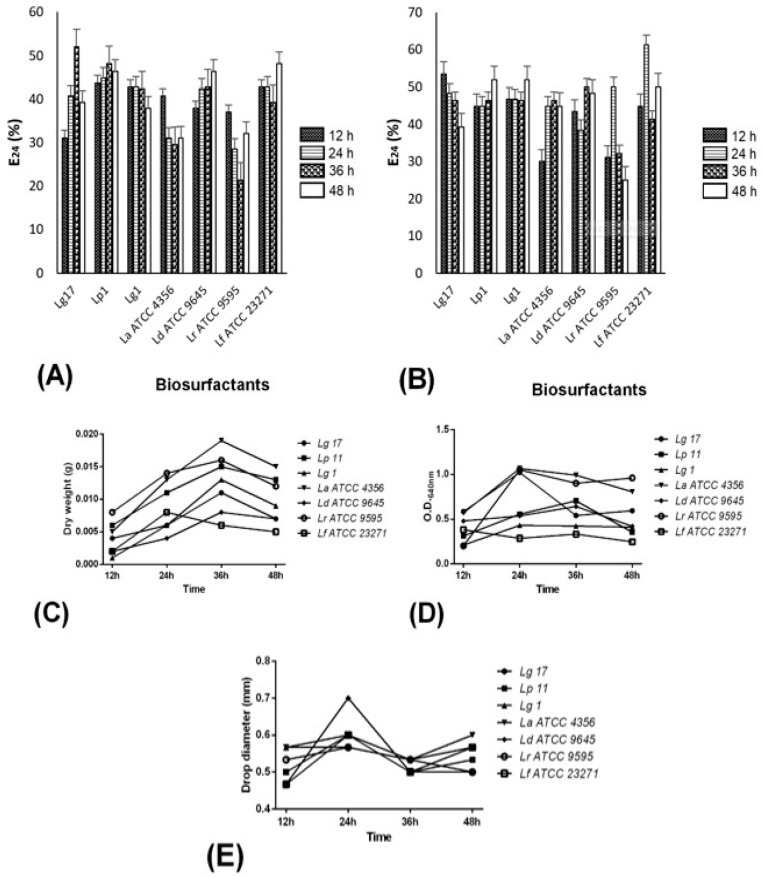
Emulsification activities, shown by *Lactobacillus* spp., produced biosurfactants expressed as an emulsification index (E_24_) and biosurfactants’ dry-weight measurement, biomass concentration, and drop-collapse assay. (**A**) Emulsification indices (E_24%_) evaluated using hexane as the organic phase. (**B**) Emulsification indices (E_24%_) evaluated using toluene as the organic phase. (**C**) Dry-weight measurement (g). (**D**) Biomass concentration (OD_640 nm_) of biosurfactants. (**E**) Biosurfactant drop-collapse assay. Results are shown over time (12, 24, 36, and 48 h) and represent the average of three independent experiments. Lg—*Lactobacillus gasseri*; Lp—*Lactobacillus paracasei*; La—*Lactobacillus acidophilus*; Ld—*Lactobacillus debrueckii*; Lr—*Lactobacillus rhamnosus*; Lf—*Lactobacillus fermentum*. Letter (**B**): *p* < 0.05, in relation to hexane; *p* > 0.05, in relation to incubation time.

BS production was evaluated by the emulsifying activity (E_24_). The strains that gave an emulsifying layer after 24 h were considered to be BS producers. Among the 20 *Lactobacillus* samples tested, four reference strains and three clinical isolates were BS producers by using hexane (Figure 1A). The following BS properties were evaluated: emulsification activity, dry weight, OD_640_ measurements, and drop collapse test. 

The emulsifying activity, designated as the emulsification index (E_24_), was evaluated using toluene or hexane as an organic solvent. According to the hydrophobic substrates evaluated, E_24_ values varied by *Lactobacillus* BS. In general, higher E_24_ values were obtained using toluene (*p* < 0.05) (Figure 1B). The highest E_24_ value (53% in 12 h), among vaginal *Lactobacillus* isolates, was obtained by Lg17 (*Lactobacillus gasseri* 17) in the presence of toluene (Figure 1B). The BS produced by Lp11 (*Lactobacillus paracasei* 11) and Lg1 only emulsified toluene solvent at values greater than 50% in 48 h (Figure 1B). There were no significant differences in BS production by any species in relation to time (*p* > 0.05). Using hexane, Lg17 reached an E_24_ of 52% in 36 h of fermentation. Lp11 and Lg1 had E_24_ values under 50% in all incubation times (Figure 1A). For the reference strains, the highest E_24_ toluene values of 50% and 61.2% were obtained for *L. rhamnosus* ATCC 9595 and *L. fermentum* ATCC 23271, respectively, in 24 h of fermentation (Figure 1B). An E_24_ value of 50% for *L. debrueckii* ATCC 9645 was only obtained by 36 h of incubation. In the presence of hexane, all E_24_ values were under 50% for every strain, with emulsifying indices from 31% to 48%. The E_24_ values for *L. rhamnosus* ATCC 9595 and *L. acidophilus* ATCC 4356 were smaller at most fermentation times in the presence of any solvents used (Figure 1A,B). The E_24_ toluene value of *L. rhamnosus* ATCC 9595 was smaller than the other reference strains (*p* < 0.05) (Figure 1B).

Depending on the isolate incubation time, dry-weight values varied significantly (*p* < 0.05). All samples showed a dry-weight gain up to 36 h, followed by a decline, except for *L. fermentum* ATCC 23271, which began to decline after 24 h. Regarding the reference strains, *L. rhamnosus* ATCC 9595 had a significantly higher dry-weight measurement in relation to *L. acidophilus* ATCC 4356, but production did not differ in relation to *L. debrueckii* ATCC 9645 or *L. fermentum* ATCC 23271 (Figure 1C). Among clinical samples, Lg1 had a significantly higher dry weight in relation to the other strains (*p* < 0.05) but did not differ in relation to the reference samples (Figure 1C).

Regarding incubation time, OD values for the different *Lactobacillus* strains varied significantly (*p* < 0.05). OD quantification showed that Lg1, Lg17, *L. rhamnosus* ATCC 9595, and *L. acidophilus* ATCC 4356 presented higher values in the first 24 h, which declined shortly after. Lg1 values remained nearly stable after the first 24 h. Lp11*, L. debrueckii* ATCC 9645, and *L. fermentum* ATCC 23271 showed higher OD values at 36 h (Figure 1D). All BS droplets resulted in a collapsed droplet in the drop-collapse test. Higher values were obtained in 24 h, and *L. debrueckii* ATCC 9645 had the highest value (0.7 cm) (Figure 1E).

### 2.4. Surface Tension Determination

Considering the surface tension (ST) value for PBS, all *Lactobacillus* strains showed ST-reducing activity. Table 4 shows ST values (mN/m) of different *Lactobacillus*-derived BSs. PBS ST was 71.9 mN/m. The highest ST reduction was obtained by Lg1, which reduced the value from 70.9 mN/m (PBS) to 49.7 mN/m.

### 2.5. BS Interference on C. albicans Adhesion

**Figure 2:**
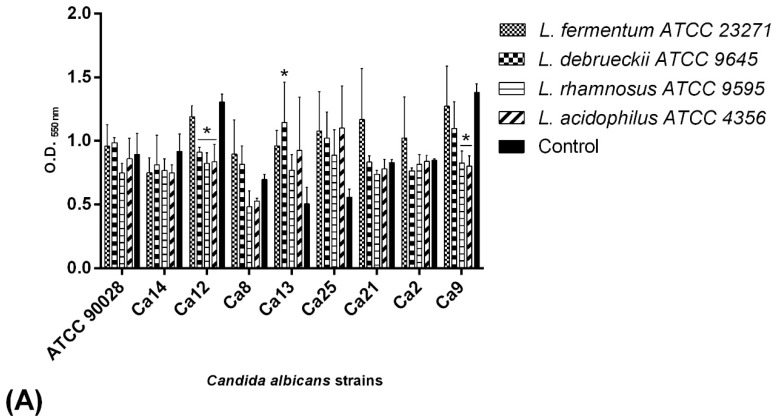

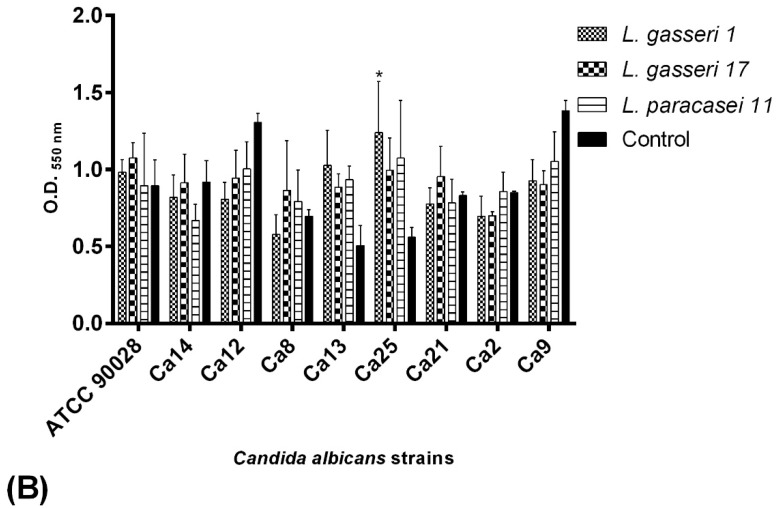
Interference of *Lactobacillus* biosurfactants on *Candida* adhesion to polystyrene. (**A**) *Lactobacillus* reference strain biosurfactants. (**B**) Biosurfactants of vaginal *Lactobacillus*. The results were expressed as absorbance values at OD_550 nm_ and compared to adhesion without *Lactobacillus* biosurfactants (control value). Statistical significance was determined at *p* < 0.05 *. Ca—*Candida albicans*.

BSs of reference strains *L. rhamnosus* ATCC 9595 and *L. acidophilus* ATCC 4356 were able to decrease the adhesion of most *Candida* studied (78%). However, interference was significant in the Ca9 and Ca12 isolates (*p* < 0.05) with inhibition values of 42% and 36%, respectively. BSs of *L. debrueckii* ATCC 9645 and *L. fermentum* ATCC 23271 decreased the adhesion of three *Candida* isolates, with the latter interfering significantly in adhesion of the Ca12 isolate (Figure 2A). Lg1 BSs decreased the adhesion in 67% of *Candida* isolates. The BSs of Lp11 and Lg17 isolates decreased the adhesion of four and three *Candida* isolates, respectively (Figure 2B). The interference of both Lg1 and Lg17 were significant in relation to the Ca9 isolate (43% reduction). All BSs decreased the adhesion process of the Ca12 isolate, whereas 67% of BSs decreased the adhesion of Ca9 and Ca14 isolates. 

All BSs, except those of Lp11, increased the adhesion of Ca13 and Ca25 isolates in relation to the control. BS from *L. fermentum* ATCC 23271 also increased the adhesion capacity of *C. albicans* isolates ATCC 90028, Ca8, Ca21, and Ca2. The product of *L. debrueckii* ATCC 9645 also enhanced the adhesion of *C. albicans* ATCC 90028 and Ca8. BS from the clinical isolate Lg17 increased the adhesion of *C. albicans* ATCC 90028, Ca8, and Ca21.

### 2.6. C. albicans Biofilm Formation Ability

**Figure 3:**
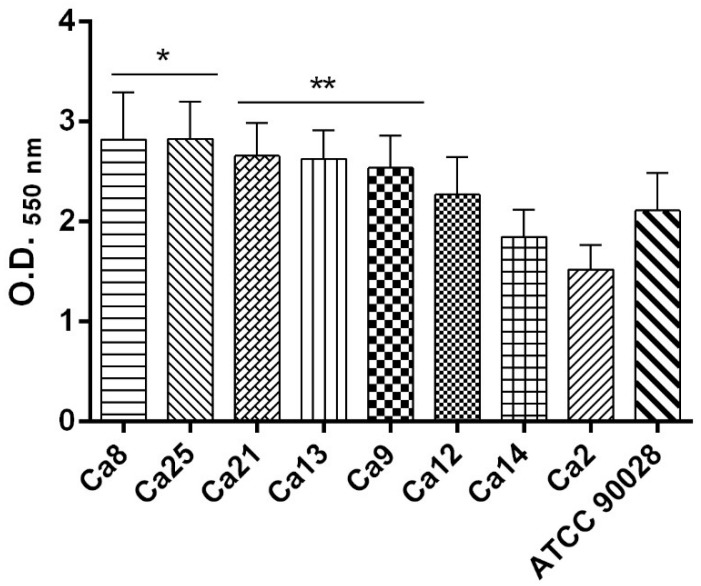
Biofilm formation by *C. albicans* isolates evaluated in YNB (Yeast Nitrogen Base) culture medium supplemented with 100 mM glucose during a 48 h period. Biofilm mass was quantified by the crystal violet staining method. Results are expressed as absorbance values at OD_550 nm_. Ca—*Candida albicans.* Symbols * and ** means statistically different values in relation to the reference strain *C. albicans* ATCC 90028 (*p* < 0.05).

Quantification of the biofilm formation of *C. albicans* isolates by the crystal violet method revealed that all the isolates tested were strong biofilm producers (Figure 3). However, biofilm formation ability varied by strain. *C. albicans* Ca8 displayed the highest biofilm formation capacity, with a mean OD_550 nm_ value of 2.823. On the other hand, *C. albicans* Ca2 strain had the lowest mean OD_550 nm_ value of 1.523. These strains gave statistically different values to the reference strain, *C. albicans* ATCC 90028, which had an OD_550 nm_ value of 2.111.

### 2.7. BS Interference in the Biofilm Formation of C. albicans Isolates

#### 2.7.1. Co-Incubation Assay

**Figure 4:**
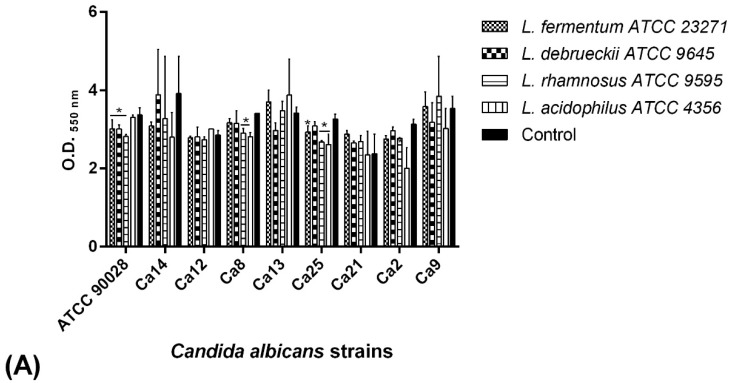

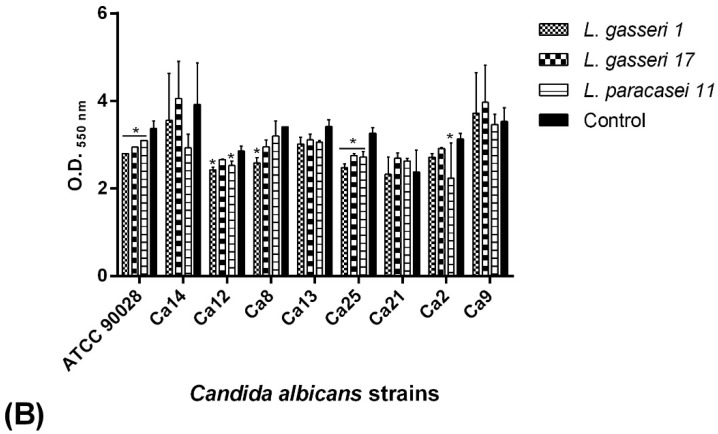
The effect of *Lactobacillus*-derived biosurfactants on *C. albicans* biofilms by the co-incubation assay. (**A**) *Lactobacillus* reference strains. (**B**) *Lactobacillus* clinical isolates. Results are shown as the optical density of the biofilm mass at 550 nm and represent the mean and standard deviation (error bars) of three independent experiments. * *p* < 0.05 for comparison between the untreated and treated groups. Ca—*Candida albicans.*

BSs produced by *Lactobacillus* reference strains inhibited biofilm formation of all clinical *Candida* isolates at different levels. The most efficient BSs were those produced by *L. rhamnosus* ATCC 9595 and *L. acidophilus* ATCC 4356, which decreased biofilm formation by 30% and 35%, respectively (Figure 4A). The BSs produced by the three clinical *Lactobacillus* strains were also able to inhibit *C. albicans* biofilms at varying levels, depending on the isolate. The BSs produced by Lg1 and Lp11 were the most efficient, reaching reduction values of 25% and 28%, respectively (Figure 4B).

#### 2.7.2. Pre-Incubation Assay

**Figure 5:**
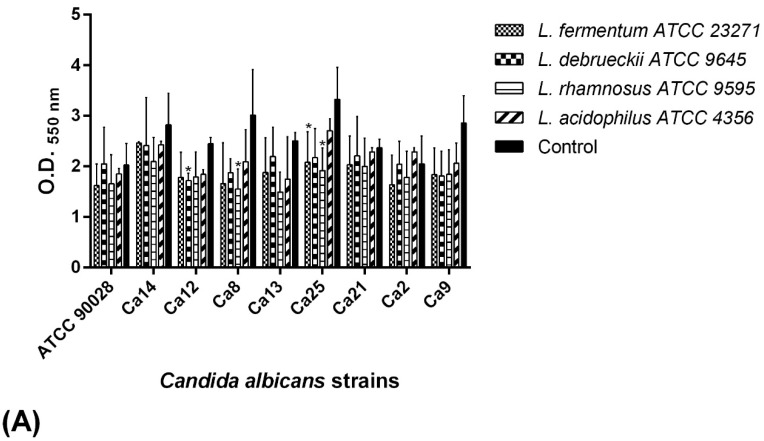

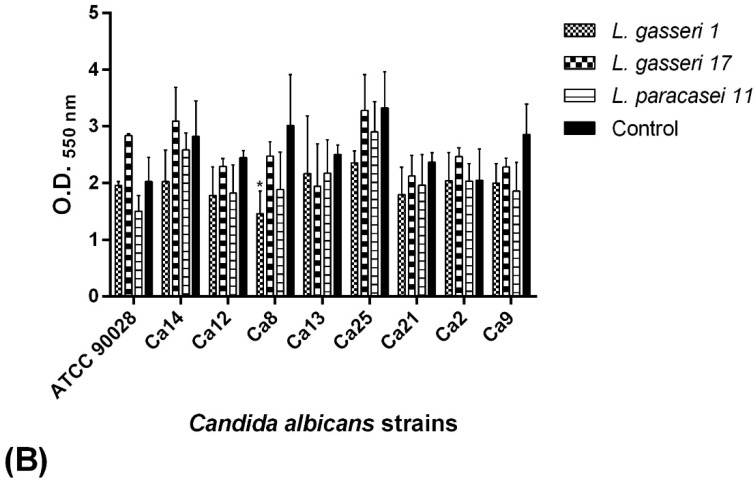
The effect of *Lactobacillus*-derived biosurfactant on *Candida albicans* biofilm by pre-incubation assay. (**A**) *Lactobacillus* reference strains. (**B**) *Lactobacillus* clinical isolates. Results are shown as absorbance values of the biofilm mass at 550 nm and represent the mean and standard deviation (error bars) of three independent experiments. * *p* < 0.05 for comparison between the untreated and treated groups. Ca—*Candida albicans.*

In the pre-incubation assay, the microplates were previously sensitized with BS. All BSs produced by the reference and clinical *Lactobacillus* strains were able to decrease biofilm formation of the tested *C. albicans* strains at varying levels (Figure 5). In this study, the most efficient BSs were those of *L. rhamnosus* ATCC 9595, which reached 44% and 50% biofilm formation reduction in Ca13 and Ca8, respectively, and *L. fermentum* ATCC 23271, which reached 40% and 47% reduction against Ca23 and Ca8, respectively. Among the clinical *Lactobacillus*-produced BSs, Lg1 and Lp11 decreased the biofilm formation of all *Candida* strains. Lg1 and Lp11 BSs decreased the biofilm of *C. albicans* Ca8 by 46% and 41%, respectively (Figure 5B).

Table 5 summarizes the results obtained for BS-producing *Lactobacillus* strains regarding the properties and potential of their BS. *Lactobacillus* from asymptomatic women had greater anti-adhesive and antibiofilm effects than *Lactobacillus* from a patient with clinically suspected VVC.

### 2.8. Antifungal Susceptibility Testing

The susceptibility profile was determined only for clinical isolates of C. albicans, which was the most frequent species. Of the nine clinical isolates, five (55.6%) were considered S to fluconazole (FLC), while four (44.4%) strains had a minimum inhibitory concentration (MIC) of 16 or 32 μg/mL. Regarding itraconazole (ITC), only two (22.2%) were considered S, and four (44.4%) were dose-dependently susceptible (DDS). All isolates were considered susceptible to amphotericin B (AMB). The reference strain (C. albicans ATCC 90028), used as a control, was susceptible to all antifungal agents (Table 6).

## 3. Discussion

*Lactobacillus* species are responsible for maintaining a healthy vaginal environment, providing a barrier to the colonization of pathogenic organisms, and inhibiting the exacerbated growth of commensal microorganisms [17]. In the present study, some strains of *Lactobacillus* showed antagonistic and antivirulence activity against *C. albicans*, including reference strains and clinical isolates. Among these strains, *L. acidophilus* ATCC 4356, *L. rhamnosus* ATCC 9595, and the clinical isolate Lp11 were able to inhibit all *C. albicans* strains tested. This preliminary analysis demonstrated an antifungal activity that might be due to one of the compounds produced by *Lactobacillus* spp. In vitro studies have reported the antimicrobial potential of *Lactobacillus* spp. against *Candida* [2,31,32,34,35,36]. Probiotics, including those of the genus *Lactobacillus*, exert antimicrobial activity through the production of various substances, such as organic acids, hydrogen peroxide, bacteriocins, antimicrobial molecules, and BSs—all of which can prevent the growth of potential pathogens [15,37].

Since probiotic LAB can produce BSs that yield in vivo defense properties against pathogen colonization, the ability of *Lactobacillus* isolates to produce BSs were verified. The emulsifying activity (E_24_), monitored during *Lactobacillus* growth in MRS broth, was used to determine BS production. Seven *Lactobacillus* strains were considered BS producers, three clinical isolates (Lg1, Lp11, and Lg17) and four reference strains (*L. fermentum* ATCC 23271, *L. rhamnosus* ATCC 9595, *L. debrueckii* ATCC 9645, and *L. acidophilus* ATCC 4356). Some of the emulsifying indexes were as high as those found by other authors for BS produced by other *Lactobacillus* species [34,35].

Different emulsification activities were obtained depending on the concentration of BSs produced and the hydrophobic substrates used in the assays. The emulsifying activity can vary depending on the organic phase chemical structure of both the BS and the emulsion [38]. Most BSs showed substrate specificity, presenting different rates of solubilization or emulsification of different hydrocarbons. In this work, emulsifying indices from 31% to 48% were obtained with hexane for the *Lactobacillus* strains that produced BSs. These index values were much smaller when compared to those obtained for the same strains against toluene. BS production in the presence of toluene as a hydrocarbon implies that the BS-producing strain utilizes various toluene components as substrates for BS production [39], thus obtaining higher concentrations of BS [40]. This shows that the choice of solvent is important for obtaining BSs with efficient emulsification properties, which are critical for promising BSs and their applications [25]. This BS–substrate specificity was also observed by other authors [34,38,41]. Besides, all BSs produced by *Lactobacillus* strains showed good ST reducing activity. BSs decreased PBS ST from 70.91 to 49.34–64.99 mN/m. The highest ST reduction was obtained for Lg1. ST acted as an indicator of surface-related properties of surfactants, such as washability and wetting. Besides, the potential of a microbial surfactant is determined by its ability to reduce the surface tension of a production medium. The ability of a biosurfactant to reduce surface and interfacial tensions determines its functionality and effectiveness. Isolates capable of reducing the ST of distilled water from 72 to 35 mN/m, or of the medium to ≤ 35 mN/m, can be considered strong biosurfactant-producing microbes [25,39].

Currently, BSs are widely used in industrial applications, mainly in heavy metal removal from contaminated soil [41] or crude oil recovery [42]. However, due to surfactant action at interfaces that modify hydrophobic characteristics, BSs have a potential role in preventing microorganism-related diseases, and, therefore, could significantly impact public health [43]. For example, medical instruments made of silicone latex or inox have highly hydrophobic, easily colonized surfaces that favor the formation of biofilms by pathogens, such as yeasts [44]. Additionally, bacterial and yeast strains have demonstrated the ability to colonize hydrophobic silicone rubber surfaces [45]. Application of *Lactobacillus* BSs could disturb microbial adhesion and desorption processes by interfering with hydrophobicity [25]. Our results support these applications.

Some BSs, such as sophorolipids, have also been used for skin treatment, acting as agents for fibrinolysis, desquamation, depigmentation, and macrophage activation [46]. Rhamnolipids, another kind of BS, are used in low concentrations (0.1%) for the treatment of ulcers and burns [47,48]. Although we do not have preliminary information regarding the chemical nature of the BSs tested in our study, the results suggested that the BSs might have similar uses; however, further research is necessary to confirm this and the composition of the BSs to be used.

The scientific world possesses little knowledge regarding the chemical nature of *Lactobacillus* BS, but research has already reported extensive variability within this compound group [19,38,40]. For instance, Rodrigues et al. [19] verified that *Lactococcus lactis* produced BSs composed of glycoproteins with glucose, rhamnose, fucose, and mannose. Morais et al. [38] found a great percentage of galactose and glucose in the chemical composition of *L. jensenii*_6A_ and *L. gasseri* P_65_. BS diversity in chemical structure (hydrocarbon composition) and carbohydrate, protein, and lipid concentrations may explain the interference variations observed between BSs on the adhesion ability of *Candida* in this study.

Assays to verify the inhibition potential of BSs on adhesion and biofilm formation showed that they were able to decrease the adhesion of the *C. albicans* strains tested, highlighting the reference strains *L. rhamnosus* ATCC 9595 and *L. acidophilus* ATCC 4356 and the clinical isolate Lp11. Some BSs, such as *L. debrueckii* ATCC 9645, showed both negative and positive interference in the adhesion processes of *Candida* isolates. Studies suggest that BSs interfere with biofilm formation, modulating surface interaction, and inhibiting the adhesion process. As previously mentioned, BSs can adsorb to surfaces by reorienting polar and nonpolar groups according to the hydrophobicity of the surface. This interaction between BSs and surface substrates alters the surface hydrophobicity, thereby intensifying or reducing the surface adhesion ability of *Candida* spp. [22]. The results obtained here, especially those in the pre-incubation assay, support this property of BSs. In this way, the in vitro model of adherence and biofilm formation used in this study were very informative in relation to understanding BS antibiofilm activity. *Lactobacillus* BSs from both reference and clinical strains disrupted the biofilm of all tested microorganisms at different levels in the co-incubation experiment. However, the best results were obtained in the pre-incubation assay, in which the microplate was previously sensitized with BSs, and all BS produced by reference and clinical strains of *Lactobacillus* were able to decrease the biofilm formation of the tested *C. albicans* strains to a high degree. For instance, the BS of *L. rhamnosus* ATCC 9595 reached values of 44% and 50% reduction in biofilm formation of Ca13 and Ca8, respectively. *L. fermentum* ATCC 23271 reached 40% and 47% reduction against Ca23 and Ca8, respectively. Among the clinical lactobacilli*,* Lg1 BS was able to decrease *C. albicans* Ca8 biofilm by 46%, and Lp11 achieved an inhibition percentage of 41% against the Ca8 isolate. The interference of *L. acidophilus* ATCC 4356 in the formation of *C. albicans* biofilm had previously been demonstrated by Vilela et al. [49]. These authors observed that filtered supernatant from a culture of *L. acidophilus* ATCC 4356 cells was able to inhibit the biofilm formation of *C. albicans* ATCC 18804.

Our data showed that Lp11 was the clinical isolate that displayed the best anti-*Candida* activity, although this property could not be attributed to BS alone, even though Lp11 showed the best emulsification index and ST value. Certainly, the anti-*Candida* activity shown by Lp 11 was due to another compound or to a synergistic combinatory action of several compounds, including BS. Furthermore, the results showed that the action of BS was most likely related to the anti-adhesive and anti-biofilm action against *Candida*.

LAB interfere in the colonization of pathogens through several mechanisms. The competition for adhesion sites, together with the secretion of BSs, is a well-known mechanism to hinder the establishment of vaginal pathogens [22,50]. The reduction of pathogen colonization to surfaces through the use of BSs produced by LAB has been described for several surfaces, including metal [46], silicone and voice prostheses [21,22,51], and glass [51], as well as other surfaces [23,52]. Results demonstrating the antibiofilm activities of *Lactobacillus* BSs support the use of BSs as a protective film for the surfaces of hospital devices, such as catheters, to prevent contamination or *Candida* infection. Taking the results as a whole, as Lg1 and Lg17 showed lower anti-*Candida* activity, their effects appear to be more associated with anti-adhesive and anti-biofilm activities. In contrast, Lp11 shows excellent anti-*Candida* activity, although our findings indicated that other compounds besides BS would probably be involved in this function and that Lp11 BS would have greater involvement in the anti-biofilm activity. 

Taking into account the anti-adhesion and antibiofilm activities presented by BS and data from the scientific literature [19,38], different types of carbohydrates or certain proteins could contribute to the chemical composition of these *Lactobacillus*-produced biosurfactants. Our results also drew attention to the possibility of intravaginal administration of pharmaceutical formulations containing BS for prevention or treatment of vaginal *Candida* infections. This study provided useful insights into the potential uses and applications of BSs; however, BSs need to be purified and characterized due to their varied compositions for an improved understanding of their anti-*Candida* and antivirulence activity. To be considered safe for use in vivo, the safety of BS should be investigated by using cytotoxicity assays.

VVC is often difficult to treat and is recurrent in most cases [13]. In this context, the drug susceptibility assays revealed that many *Candida* isolates of this study were considered DDS or R to ITC (77.7%) and FLC (44.4%). These findings point to a phenomenon of increased resistance against conventional antifungal drugs and reiterate the need to identify new potential alternatives against these pathogens.

## 4. Materials and Methods 

### 4.1. Patients and Ethical Aspects

The Ethics Committee of the CEUMA University approved the research protocols (number 813.402/2014), and all methods were performed per the relevant guidelines and regulations. Samples were collected from 50 patients at the Hospital da Mulher, São Luís, Maranhão, Brazil, during preventive tests for cervical cancer, after signing an informed consent form. Informed consent was obtained from all subjects. The exclusion criteria included women who had used antifungal medication (oral and/or vaginal) or antibiotics in the last 30 days, or who had diseases that affected the immune system.

### 4.2. Statement

All experiments and methods were performed per relevant guidelines and regulations. All experimental protocols were approved by The Ethics Committee of the CEUMA University, specifically vaginal secretion samples collection (and relevant protocols) (813.402/2014). All subjects gave their informed consent for inclusion before they participated in the study, and all methods were carried out per the Declaration of Helsinki and guidelines/regulations of CEUMA Ethics Committee.

### 4.3. Isolation and Identification of Vaginal Microorganisms

Cervicovaginal sampling was performed using sterile swabs, which were immersed in sterile tubes containing BHI (Brain Heart Infusion; Difco Laboratories Inc., Detroit, MI, USA) or MRS (Man, Rogosa, and Sharpe broth; Difco, Detroit, MI, USA). Samples in BHI and MRS were placed at 37 °C for 24 h and then seeded in MRS agar and SDA medium (Sabouraud Dextrose Agar; Difco Laboratories Inc., Detroit, MI, USA) for 48 h. MRS agar plates were incubated under anaerobic conditions for *Lactobacillus* spp. isolation (Anaerobic System, Probac LTD, São Paulo, Brazil). SDA was used for *Candida* spp. isolation. A polymerase chain reaction (PCR) with multiple specific primers (multiplex PCR) was used for yeast identification (Supplementary Material S1) and partial sequencing of 16S subunit rDNA (Supplementary Material S2) for *Lactobacillus* identification.

In addition to clinical isolates, reference strains were also included in this study (Supplementary Material S3).

### 4.4. Antagonism Assay

The antagonism assay was performed by the overlay method [31]. Cultures of *Lactobacillus* spp. were incubated under anaerobic conditions at 37 °C for 24 h in MRS broth supplemented with 0.25% L-cysteine (MRS-CYS, Sigma-Aldrich, St. Louis, Missouri, USA). Bacterial samples were standardized at 0.1 at OD_600 nm_, and then 10 μL of each sample was plated on MRS-CYS agar and incubated under the same conditions. After this period, a 2 mm layer of SDA was added, and the *C. albicans* standard inoculum (1 × 10^7^ cells/mL) was seeded on top. Petri dishes were incubated at 37 °C for 24 h under aerobic conditions, and the subsequent measurements for zones of inhibition were taken.

### 4.5. BS Production by Lactobacillus Spp.

Initially, a small-scale culture of all *Lactobacillus* spp. was carried out to determine bacteria capable of producing BSs. Once those bacteria were identified, large-scale production of BS was carried out [53]. First, a colony isolated from each strain was added to 10 mL of MRS-CYS broth and incubated for 16 h at 37 °C under anaerobic conditions. Then, a 4 mL aliquot was inoculated into 400 mL of MRS-CYS and incubated (anaerobic conditions, 48 h, 37 °C) for large-scale BS production. After the incubation period, a centrifugation step (10,000× *g*, 5 min at 10 °C) was used to harvest the cells, which were then washed and suspended in PBS solution (phosphate-buffered saline; pH 7; 15 mL of PBS for each 100 mL of culture). The suspension was kept at room temperature for 2 h with gentle stirring for biosurfactant release and then centrifuged (1904× *g* for 15 min). The supernatant was collected, filtered using a 0.22 mm-pore-size filter (Merck KGaA, Darmstadt, Germany), lyophilized, and stored at −20 °C. During the 48 h incubation period, aliquots were removed every 12 h for a dry-weight measurement, an OD_640 nm_ check, and drop-collapse and emulsifying activity tests. 

#### 4.5.1. Emulsifying Activity Determination

Using either toluene or hexane as the hydrophobic substrate, the emulsifying activity (E_24_) was measured to confirm BS production [35]. Two milliliters of toluene or hexane were added to an equal volume of sample and then vortexed at high speed for 2 min and incubated for 24 h at room temperature. Emulsification indices (E_24_, _%_) were the percentage of the height of the emulsifying layer (mm) divided by the height of the total layer (mm).

#### 4.5.2. Dry Weight and OD_640 nm_ Measurements

For dry-weight evaluation, an empty microtube was first weighed, and then 1 mL of sample was added. Microtubes were centrifuged, and the cells were dried at 60 °C and weighed again. Dry-weight values were obtained from the equation,

*dw* = *Mf* − *Mi*

where *dw* was the dry weight (g), *Mf* was the final weight of the microtube (g), and *Mi* was the initial weight of the microtube (g).

Biomass values were recorded for all samples during the 12, 24, 36, and 48 h culture intervals by measuring the optical density of each culture at 640 nm using a spectrophotometer (Biotek, Goiânia, Brazil).

#### 4.5.3. Drop-Collapse Method

A drop-collapse test was performed to verify whether BSs were able to reduce the surface tension (ST) between an aqueous solution and hydrophobic surfaces. Tests were performed in triplicate, and PBS was used as a control following recommendations [35]. Ten microliters of BS were added to a polystyrene plate well, and the spreading/flattening of the droplet on the polystyrene surface was monitored. The droplet was allowed to dry, and the diameter of the dried droplet was recorded. 

#### 4.5.4. ST Determination

BS ST values were measured by the plate method using a KRUSS tensiometer (K10T model, KRUSS, Hamburg, Germany) at room temperature to determine the relationship between BS concentration and ST. First, LAB cells were recovered by centrifugation (10,000 × *g*, 10 min, 4 °C), then washed twice with PBS and resuspended in PBS. To calibrate the tensiometer, the ST measure was performed twice with distilled water. All ST measurements were performed in triplicate and averaged. Sterile PBS was used as a control.

### 4.6. Selection of C. albicans Biofilm Producers

Yeast Nitrogen Base (YNB) standardized inoculum (200 μL; 1 × 10^7^ cells/mL) and 100 mM glucose were added to 96-well flat-bottom microplates. The test was performed with eight replicates, and the wells, containing only culture medium, were used as controls. Microplates were incubated for 90 min (adhesion period). Cells were washed with PBS, followed by the addition of 200 μL of fresh medium and subsequent incubation for 48 h at 37 °C under aerobic conditions. The culture medium was changed every 24 h. For biofilm quantification, the medium was removed, and wells were washed with PBS. Cells were fixed with 200 μL of 80% ethanol for 15 min. The ethanol was removed, and plates were dried at room temperature and stained with 200 μL of crystal violet (2%) for 20 min. PBS wash removed any excess stain. Crystal violet bound to the adherent microorganisms was resolubilized with 150 μL glacial acetic acid solution (33%) added to each well, and 150 µL of the resultant mixture was transferred to new microplates. Microplate absorbance was measured at 550 nm [52].

### 4.7. BS Interference on C. albicans Adhesion

Two hundred microliters of each crude 48 h BS in PBS (5 mg/mL) were added to 96-well microplate. The wells, containing only PBS, were used as controls. After 16 h of incubation at 4 °C, microplates were washed twice with PBS. Then, a 200 μL aliquot of yeast suspension (0.38 at OD_550 nm_) was added and incubated for 4 h at 4 °C. Tests were performed in triplicate on two different occasions. Wells were washed three times with PBS and then stained with crystal violet (Section 4.6). Attached cells were quantified using a microplate reader (Biotek, Goiânia, Brazil) at 550 nm. Adhesion inhibition percentages for various BSs for each microorganism were determined as:

% Adhesion Inhibition = (1 − (A_BS_)/A_0_) × 100

where A_BS_ was the absorbance of the well treated with BS, and A_0_ was the absorbance of the control well without BS [25].

### 4.8. BS Interference on C. albicans Biofilm Formation

Assays were performed under both pre-coating and co-incubation conditions [52]. In the pre-coating experiments, prior sensitization of the 96-well microplate was made by adding 200 μL of crude 48 h BS in PBS (5 mg/mL) and incubating for 24 h at 37 °C. The wells, containing only water, were used as a control. Plates were washed twice with PBS. *C. albicans* inoculum (200 μL; 0.38 at OD_550 nm_) was added, and microplates were incubated at 37 °C for 3 h. Wells were washed twice with PBS, and 200 μL of fresh medium was added. Wells were incubated for 48 h at 37 °C, and the medium was changed every 24 h.

In co-incubation experiments, *C. albicans* inoculum and BS were placed together in the wells in a 1:1 ratio and were incubated for 3 h under the same conditions as the previous test. After the washing process, wells were again filled with new medium and BS in the same ratio. Incubation conditions were maintained. Biofilm production was quantified as in Section 4.6. 

Both tests were performed with three replicates and a control with no BS interference. For interpretation, the absorbance of each well was measured at 550 nm and compared with that of the control well.

### 4.9. Antifungal Susceptibility Testing

Susceptibility profiles of *C. albicans* isolates were established in relation to itraconazole (ITC), fluconazole (FLC), and amphotericin B (AMB), according to CLSI (Clinical Laboratory Standard Institute) [54]. Diluted antifungals were incubated in 96-well microplates with *C. albicans* isolates (2.5 × 10^3^ CFU/mL) at 35 °C for 48 h. Vehicle (RPMI)-treated wells without fungal isolates were used as negative controls. All tests were performed in triplicate on three different occasions. The MIC (minimum inhibitory concentration) of each antifungal was considered the lowest concentration at which no fungal growth was observed. Breakpoints were those defined by CLSI [54]. *C. albicans* ATCC 90028 was used as a control. 

### 4.10. Statistical Analysis

Data were analyzed by IBM SPSS Statistics 20 (2011). Initially, a chi-square test of independence (χ^2^) was used to evaluate the association of *Lactobacillus* and *Candida* spp. present in the vaginal microbiota of healthy and VVC women. Subsequently, normality tests of all numerical variables (inhibition halo, biofilm, BS production, BS on adhesion, BS on biofilm) were performed using the Shapiro–Wilk test followed by parametric tests (ANOVA, Tukey, Dunnett, ANCOVA, and Pearson’s correlation). The effect of *Lactobacillus* spp. was evaluated by ANOVA. In all tests, the level of significance (α) was 5%.

## 5. Conclusions

In conclusion, this study showed *Lactobacillus* strains with significant anti-*Candida* activity by inhibiting the growth of vaginal isolates. Some *Lactobacillus* strains produced BSs with high emulsifying indexes. *Lactobacillus* spp. produced BSs that exhibited considerable anti-adhesion and antibiofilm activities against *C. albicans*. Although the BSs in this study were not characterized, the results were promising and indicated future medical applications for analyzed BSs in the prevention and treatment of *Candida* infections. We highlighted the clinical isolate *L. gasseri* Lg1 and *L. paracasei* Lp11, both from vaginal secretions of asymptomatic patients, as promising sources of medically applicable BSs.

## Figures and Tables

**Table 1:** Identification of *Candida* and *Lactobacillus* isolates from vaginal microbiota of asymptomatic women (control group) and women with clinical suspicion of VVC from a public hospital in São Luís, Maranhão, Brazil.

Patient ID	Condition	*Candida* Identification	*Lactobacillus* Identification	
		Multiplex PCR	16S rRNA Sequencing (% homology)	No. Access
V2	VVC	*C. albicans*	*L. gasseri* (100%)	MK982449
V3		*C. glabrata*		
V5		*C. glabrata*		
V6		*C. glabrata*		
V8		*C. albicans*		
V10		*C. glabrata*		
V12		*C. albicans*		
V14		*C. albicans*		
V15			*L. gasseri* (100%)	MK982454
V17			*L. gasseri* (100%)	MK982456
V19		*C. glabrata*	*L. vaginalis* (99%) and *L. gasseri* (100%)	MN019109MK982457
V21		*C. albicans*	*L. vaginalis* (100%)	MK982458
V23			*L. crispatus* (100%)	MK982461
V24			*L. paracasei* (99%)	MK982460
A1	Asymptomatic		*L. gasseri* (100%)	MK982448
A4		*C. glabrata*	*L. gasseri* (97.49%)	MK982450
A7			*L. vaginalis* (99%)	MK982451
A9		*C. albicans*	*L. rhamnosus* (100%)	MK982452
A11			*L. paracasei* (99.37%)	MK982453
A13		*C. albicans*		
A16			*L. gasseri* (100%)	MK982455
A18		*C. krusei*		
A20		*C. parapsilosis*		
A22			*L. gasseri* (100%)	MK982459
A25		*C. albicans*		

Legend: VVC, suggestive for vulvovaginal candidiasis; ID = sample identification; YSARGBN locus (*Candida albicans* 5.8S ribosomal RNA gene, complete, 28S ribosomal RNA gene, 5 ‘end) was used for the identification of *C. albicans* isolates (GenBank: L47111.1), and locus AB032177 (*Candida glabrata* genes for ITS1, 5.8S rRNA), ITS2) for *C. glabrata* isolates identification (GenBank: AB032177).

**Table 2:** Antagonism test between *Lactobacillus* spp. and *Candida albicans* performed by an overlay technique on Man, Rogosa, and Sharpe (MRS) agar medium. Diameters of inhibition zone values (mm) were measured after 48 h of incubation. Inhibitory ability was dependent upon both the *Candida* and *Lactobacillus* strains tested (Tukey’s statistical test).

*Lactobacillus* ^1^	*Candida albicans* Isolates ^2^							
	Ca2	Ca8	Ca9	Ca12	Ca13	Ca14	Ca21	Ca25
*L. gasseri* V17	NZ	NZ	NZ	13 ± 0	NZ	NZ	NZ	NZ
*L. vaginalis* V19.1	10 ± 0	12 ± 1.41	12.5 ± 0.70	NZ	11 ± 0	NZ	NZ	NZ
*L. gasseri* V19.2	NZ	NZ	NZ	NZ	NZ	NZ	NZ	NZ
*L. vaginalis* V21	18.5 ± 2.12	NZ	13.5 ± 3.53	12.5 ± 0.70	15.5 ± 0.70	15.5 ± 2.12	15.5 ± 0.70	14.5 ± 2.12
*L. crispatus* V23	NZ	13 ± 1.41	11.5 ± 0.70	NZ	NZ	NZ	NZ	11 ± 0
*L. paracasei* V24	9.5 ± 0.70	13 ± 1.41	11 ± 1.41	15 ± 0	NZ	13.5 ± 0.70	NZ	11 ± 0
*L. gasseri* A1	15.5 ± 0.70	NZ	NZ	10.5 ± 0.70	NZ	14 ± 1.41	NZ	NZ
*L. gasseri* A4	NZ	12.5 ± 0.70	16 ± 1.41	NZ	14 ± 1.41	14 ± 1.41	NZ	13.5 ± 0.70
*L. vaginalis* A7	NZ	16 ± 1.41	16 ± 0	19.5 ± 2.12	22.5 ± 3.53	20 ± 0	17 ± 1.41	16.5 ± 4.94
*L. paracasei* A11	26 ± 1.41	19.5 ± 0.7	18.5 ± 2.12	13.5 ± 3.53	17 ± 8.48	21.5 ± 4.94	19.5 ± 2.12	16 ± 1.41
*L. gasseri* A16	NZ	15.5 ± 0.70	NZ	NZ	20.5 ± 2.12	13.5 ± 0.70	16 ± 0	11.5 ± 0.70
*L. gasseri* A22	NZ	NZ	NZ	NZ	12.5 ± 0.70	NZ	NZ	NZ
*L. acidophilus* ATCC 4356	21.5 ± 2.12	16 ± 0	13 ± 1.41	21.5 ± 2.12	17 ± 2.82	15.5 ± 6.36	15.5 ± 0.70	19.5 ± 0.70
*L. debrueckii* ATCC 9645	NZ	NZ	NZ	NZ	NZ	13 ± 1.41	NZ	11.5 ± 0.70
*L. fermentum* ATCC 23271	17 ± 1.41	16 ± 1.41	13 ± 0	NZ	15 ± 4.24	12 ± 1.41	12.5 ± 0.70	14.5 ± 3.53
*L. paracasei* ATCC 335	28.5 ± 2.12	17.5 ± 0.70	15 ± 5.65	NZ	17.5 ± 0.70	17.5 ± 3.53	15.5 ± 0.70	16 ± 1.41
*L. rhamnosus* ATCC 9595	22.5 ± 0.70	20 ± 0	18.5 ± 0.70	21 ± 4.24	20 ± 0	19.5 ± 0.70	25 ± 7.07	19.5 ± 0.70

Legend: NZ means no inhibition zone; ^1^ *Lactobacillus* inoculum: ~4.5 × 10^7^ CFU/mL; ^2^ *Candida* inoculum: 1 × 10^7^ cells/mL.

**Table 3:** Independent chi-square test of the proportion of inhibition of *Candida* isolates by *Lactobacillus* from patients with clinically suspected VVC and those of asymptomatic women.

% Inhibited *Candida* *albicans* Isolates	
VVC	Asymptomatic
12.5	37.5
50	62.5
0	87.5
87.5	100
37.5	62.5
75	12.5
% means: 43.75	60.42
χ^2^ = 11.26 *p* = 0.046	

**Table 4:** Surface tension values (mN/m) of biosurfactant with different *Lactobacillus* strains grown in MRS medium at 37 °C. Surface tension values were measured in PBS (Phosphate-buffered Saline) after biosurfactant recovery. The surface tension of PBS was 71.9 ± 0.1 mN/m. Results represent the average of three independent experiments ± standard deviation.

Microorganisms	Surface Tension (mN/m) ± Standard Deviation
*L. gasseri* 17	52.25 ± 0.10
*L. paracasei* 11	55.25 ± 0.07
*L. gasseri* 1	49.34 ± 0.09
*L. acidophilus* ATCC 4356	61.79 ± 0.17
*L. delbrueckii* ATCC 9645	64.46 ± 0.06
*L. rhamnosus* ATCC 9595	64.99 ± 0.06
*L. fermentum* ATCC 23271	53.85 ± 0.14
PBS	71.90 ± 0.10

**Table 5:** Principal properties and potential of biosurfactants produced by vaginal and reference *Lactobacillus* strains against *Candida* spp. Emulsification index (E_24_), Optical density (OD), and dry-weight values are those obtained within 48 h of experimentation.

Biosurfactant Properties									
*Lactobacillus*	Patients Groups	Ability to Inhibit *Candida* Growth (Antagonism Test; %)	E_24_	Dry Weight	OD_640nm_	ST	Ability to Inhibit *Candida* Adhesion (%)	Ability to Inhibit *Candida* Biofilm (Pre-Coating Assay; %)	Ability to Inhibit *Candida* Biofilm (Co-Incubation Assay; %)
*L. acidophilus* ATCC 4356	-	100	44.80	0.015	2.028	61.79	78	89	67
*L. debrueckii* ATCC 9645	-	25	48.20	0.007	1.985	64.46	44	78	78
*L. fermentum* ATCC 23271	-	87.5	50	0.005	1.898	53.85	33	100	67
*L. rhamnosus* ATCC 9595	-	100	25	0.012	2.060	64.99	78	100	67
*L. paracasei* 11	Asymptomatic	100	51.80	0.009	2.026	35.25	44	89	89
*L. gasseri* 1	Asymptomatic	37.5	51.80	0.013	1.970	49.34	67	89	89
*L. gasseri* 17	VVC	12.5	39.20	0.007	2.033	52.25	22.2	67	67

Legend: VVC: vulvovaginal candidiasis; E_24_: emulsification index in the presence of toluene; OD: optical density; ST: surface tension values (mN/m).

**Table 6:** Minimum inhibitory concentration (MIC) of *C. albicans* isolates from vaginal secretion samples of vulvovaginitis and asymptomatic women (control) groups from a public hospital in São Luís, Maranhão, Brazil.

PATIENTS GROUPS	STRAINS	SPECIES	MIC_100_ (µg/mL)		
			FLC	ITC	AMB
VVC	Ca2	*C. albicans*	8	0.5	1
	Ca8		16	8	0.5
	Ca12		4	0.5	0.25
	Ca14		8	0.062	0.5
	Ca21		8	0.125	0.5
Control	Ca9	*C. albicans*	8	1	0.5
	Ca13		16	8	1
	Ca25		16	0.25	0.5
	Ca18		32	0.25	1
-	ATCC 90028	*C. albicans*	2	0.5	0.5

Legend: VVC, vulvovaginitis group; MIC, minimum inhibitory concentration; Ca, *Candida albicans*; FLC, fluconazole; ITC, itraconazole; AMB, amphotericin B. FLC MIC values ≤ 8 μg/mL were considered susceptible (S), 16–32 μg/mL were considered dose-dependently susceptible (DDS), and ≥ 64 μg/mL were considered resistant (R). For AMB, MICs ≤ 1 μg/mL were considered S and > 1 μg/mL were considered R. For ITC, MICs ≤ 0.125 μg/mL were considered S, 0.25–0.5 μg/mL were considered DDS, and ≥ 1 μg/mL were considered R.

## Supplementary Materials

The following are available online at https://www.mdpi.com/2076-0817/8/3/150/s1, Supplementary Material S1: Describe the employed methodology for *Candida* spp. identification by multiplex PCR, Table S1: PCR multiplex primer sequences for Candida spp. identification, Supplementary Material S2: Describe the identification of *Lactobacillus* isolates by partial sequencing of 16S subunit rDNA, Supplementary Material S3: Lists the reference strains used in this work.
