# Supplementary material for: Antifungal and Antivirulence Activity of Vaginal Lactobacillus Spp. Products against Candida Vaginal Isolates
Source: Pathogens. 2019 Sep 12;8(3):150. doi: 10.3390/pathogens8030150 (PMC6789721; doi:10.3390/pathogens8030150)
Supplement: Supplementary file 1 [file pathogens-08-00150-s001.pdf]

Antifungal and antivirulence activity of vaginal *Lactobacillus* spp. products against *Candida* vaginal isolates

Camilla Itapary Santos[1], Yasmine Ramos França[1], Carmem Duarte Lima Campos[2], Maria Rosa Quaresma Bomfim[3], Bruna Oliveira Melo[3], Rodrigo Assunção Holanda[3], Vera Lúcia Santos[4], Sílvio Gomes Monteiro[5,6], Eduardo Buozi Moffa[2], Andréa Souza Monteiro[2], Cristina Andrade Monteiro[2,7*] and **Valério Monteiro-Neto[2,5]**

Supplementary Material

1.1. Supplementary material 1 - Identification of Candida spp. by multiplex PCR

DNA extraction followed the protocol proposed by Jain et al.[1], with some modifications. SDA-isolated colonies were collected and added to 600 µL of STES buffer (25 mM EDTA, 200 mM Tris, 100 mM NaCl, 1% SDS), vortexed for 2 min and heated in a water bath at 65.5°C for 10 min. Then 500 µL of CIA (24 mL chloroform, 1 mL isoamyl alcohol) were added and samples were centrifuged at 1542 × g for 5 min. Five hundred microliters of ice cold absolute ethanol were added to the supernatant and the microtubes were incubated at -20°C for 12 h. Samples were centrifuged at 1542 × *g* for 5 min for DNA precipitation and alcohol withdrawal. The precipitated DNA was washed with 500 µL of 70% ethanol and the samples were centrifuged at 1542 × *g* for 5 min. The alcohol was removed and the samples were allowed to dry at 28ºC. After drying, samples were resuspended in TE buffer (Tris-Cl 1 M, pH 7.6; EDTA 0.5 M, pH 8.0).

Candida spp. identification was done according to the methods of Luo and Mitchell [2] with modifications. Initially, species identification was made using specific *Candida* primers designed from the ITS region called ITS1 and ITS4. Identification at the species level was done with the following pairs of primers: CALB1/CALB2 for *C. albicans*, CTR1/CTR2 for *Candida tropicalis*, CPA2/CPA3 for *Candida parapsilosis*, CGL1/CGL2 for *Candida glabrata*, and CKR2/CKR3 for *Candida krusei* (Supplementary table S1). Two PCR multiplex panels were performed. The first set contained primers for the identification of *C. glabrata*, *C. tropicalis,* and *C. parapsilosis*, and the second contained primers for the identification of *C. albicans* and *C. krusei*. Each reaction mixture (20 µL total volume) contained genomic DNA (~1 ng), Tris-HCl (20 mM, pH 8.4), KCl (50 mM), $MgCl_2$ (1.5 mM), deoxyribonucleotide triphosphate (0.2 mM of each), *Taq* DNA polymerase (0.5 U) (Invitrogen Life Technologies, Carlsbad, CA, USA), and a specific pair of primers (0.5 µM for ITS1/ITS4; 0.7 µM for CGL1/CGL2; 0.4 µM for CTR1/CTR2; 0.6 µM for CPA1/CPA2; and 0.4 µM for CKR2/CKR3). Conditions for PCR amplification were 5 min denaturation at 96°C, followed by 40 cycles of 94°C for 30 s, 58°C for 30 s, 72°C for 30 s, and a final extension step of 72°C for 15 min. Ten microliters of the final product were used for 2% agarose gel electrophoresis for 2–3 h. Bands of DNA were visualized after ethidium bromide staining in UV transilluminator (Loccus Biotecnologia, San José, Brazil).

Table S1. PCR multiplex primer sequences for *Candida* spp. identification.

Primers	Sequence	Amplicon	References
Genus *Candida*	F: 5′-TCC GTA GGT GAA CCT GCG G-3′ R: 5′-TCC TCC GCT TAT TGA TAT GC-3′	500–874 bp	Luo and Mitchell, 2002 [2]
CALB ½	F: 5′-TTTATCAACTTGTCACACCAGA-3′ R: 5′-ATCCCGCCTTACCACTACCG-3′	274 bp	Luo and Mitchell, 2002 [2]
CGL ½	F: 5′-TTATCACACGACTCGACACT-3′ R: 5′-CCCACATACTGATATGGCCTACAA-3′	430 bp	Luo and Mitchell, 2002 [2]
CPAR 3/2	F: 5′-GCCAGAGATTAAACTCAACCAA-3′ R: 5′-CCTATCCATTAGTTTATACTCCGC-3′	297 bp	Luo and Mitchell, 2002 [2]
CTR ½	F: 5′-CAATCCTACCGCCAGAGGTTAT-3′ R: 5′-TGGCCACTAGCAAAATAAGCGT-3′	355 bp	Luo and Mitchell, 2002 [2]
CKR 2/3	F: 5′-ACTACACTGCGTGAGCGGAA-3′ R: 5′-AAAAAGTCTAGTTCGCTCGG-3′	360 bp	Bougnoux, 1999

1.2. Supplementary material 2 - Identification of Lactobacillus isolates by partial sequencing of 16S subunit rDNA

Bacterial DNA extraction from isolated colonies on BHI agar was performed according to the methodology described by Pitcher et al. [3]. The concentration and purity of the obtained product were quantified in Nanodrop™ 1000 spectrophotometer (Thermo Fisher Scientific, Wilmington, DE, USA) at 260 and 280 nm. Amplification of the 16S rDNA partial sequences were performed through the universal primers for bacteria: 8F (5′-AGAGTTTGATCCTGGCTCAG-3′) and 907R (5′-CCGTCAATTCCTTTRAGTTT-3′). The amplification reaction was performed in a thermal cycler (Applied Biosystems, Foster City, CA, USA) with an initial desaturation at 94ºC for 5 min, followed by 21 cycles of desaturation at 94ºC for 1 min, annealing at 57ºC for 1 min in the first three cycles with a drop of 1ºC every two cycles until the temperature reached 49°C in the last two cycles and extension at 72°C for 3 min, finishing with a final extension at 72°C for 10 min [4]. The concentration and purity of the amplification product were quantified in a Nanodrop™ 1000 spectrophotometer (Thermo Fisher Scientific, Wilmington, DE, USA) at 260 and 280 nm and the fragment integrity of approximately 900 base pairs was analyzed by 1% agarose gel electrophoresis. The 16S rDNA region sequencing reaction was performed using BigDye® Terminator v3.1 Ready Reaction Mix (Life Technologies Corp., Carlsbad, CA, USA) and primers 8F and 907R at a concentration of 5 µmol/L The reaction product was purified and resuspended in Hi-Di™ formamide (Applied Biosystems, Foster City, CA, USA) for analysis on a 3730xl DNA Analyzer Sequencer (Applied Biosystems, Foster City, CA, USA). The sequences obtained were edited using the Sequencher 4.1.4 program and when possible, the product of the reactions using the different primers were aligned for generation of the consensus sequence and compared with sequences deposited in the GenBank database (http://www.ncbi.nlm.nih.gov/genbank/) through the BLASTn

tool. Isolates were considered the same species as in the database sequences, when values were greater than 97%.

1.3. Supplementary material 3 - Reference strains

In addition to clinical isolates, the following reference strains were used in this study: *L. debrueckii* ATCC 9645, *L. rhamnosus* ATCC 9595, *L. acidophilus* ATCC 4356, *L. fermentum* ATCC 23271, *L. paracasei* ATCC 335, *Escherichia coli* ATCC 8739 and *C. albicans* ATCC 90028. The isolates were cultivated in MRS under anaerobic conditions (*Lactobacillus* spp.) or in SDA agar (*Candida* spp.) at 37 °C, 24 h and preserved in 30% glycerol storing at −80◦C incubator.

Reference

1. Jain, P.; Khan, Z.K.; Bhattacharya, E.; Ranade, S.A. Variation in random amplified polymorphic DNA (RAPD) profiles specific to fluconazole-resistant and sensitive strains of *Candida albicans*. *Diagn. Microbiol. Infect. Dis*. **2001**, *41*, 113–119, doi:10.1016/S0732-8893(01)00292-9.
2. Luo, G.; Mitchell, T.G. Rapid identification of pathogenic fungi directly from cultures by using multiplex PCR. *J. Clin. Microbiol.* **2002**, *40*, 2860–2865, doi:10.1128/JCM.40.8.2860-2865.2002.
3. Pitcher, D.G.; Saunders, N.A.; Owen, R.J. Rapid extraction of bacterial genomic DNA with guanidium thiocyanate. *Lett. Appl. Microbiol.* **1989**, *8*, 151–156, doi:10.1111/j.1472-765X.1989.tb00262.x.
4. Marchesi, J.R.; Sato, T.; Weightman, A.J.; Martin, T.A.; Fry, J.C.; Hiom, S.J.; Dymock, D.; Wade, W.G. Design and evaluation of useful bacterium-specific PCR primers that amplify genes coding for bacterial 16S rRNA. *Appl. Environ. Microbiol.* **1998**, *64*, 795–799.
